# Supplementary material for: Spotsweeper-py: spatially-aware quality control metrics for spatial omics data in the Python ecosystem
Source: F1000Res. 2026 Sep 21;15:33. Originally published 2026 Jan 9. [Version 2] doi: 10.12688/f1000research.175162.2 (PMC13755621; doi:10.12688/f1000research.175162.2)
Supplement: Peer review history

# Reviewer report: Reviewer response for version 2

**DOI:** 10.5256/f1000research.210055.r529614

**Published:** 2026-11-11

**Authors:** Guo-Cheng Yuan, Jiazhang Cai

**Affiliations:** 1 Genetics and Genomic Sciences, Icahn School of Medicine at Mount Sinai, New York, New York, USA; 2 Icahn School of Medicine at Mount Sinai, New York, New York, USA

This paper presents a Python implementation of spotsweeper -- a method developed by the same authors for QC analysis of spatial transcriptomics data. The main idea behind spotsweeper is to detect and remove outliers using local background as opposed to data filtering using global metrics. In the previous work (and here as well), the authors showed that spotsweeper has the advantage for preserving subregions with relatively low counts while being sensitive for detecting isolated spots with poor data quality. In the original paper, spotsweeper was implemented in R. The motivation for reimplementing into Python is to be more easily adopted by Python users.  The paper is well written. I only have a few minor comments.

1. While I understand the rationale behind reimplementation, it may be worthwhile to note that R packages can be called within Python, and that there are established packages to convert data formats between R to Python. This provides an alternative way for interoperability.
2. It is somewhat surprising that the Python version uses more computing resources than R (Figure 11). It seems there is no real benefit using the Python version other than interoperability with other Python packages (such as scverse).
3. In VisiumHD analysis, most people use 8um or 16um resolution because the data is too sparse at 2um. Does the spatial resolution significantly impact the outcome of outlier detection? If so, this may create a problem for downstream analysis. Is it possible to create a uniform filter across all resolutions?

Are the conclusions about the tool and its performance adequately supported by the findings presented in the article?

Yes

Is the rationale for developing the new software tool clearly explained?

Yes

Is the description of the software tool technically sound?

Yes

Are sufficient details of the code, methods and analysis (if applicable) provided to allow replication of the software development and its use by others?

Yes

Is sufficient information provided to allow interpretation of the expected output datasets and any results generated using the tool?

Yes

Reviewer Expertise:

Computational Biology; Spatial Multi-omics Analysis

We confirm that we have read this submission and believe that we have an appropriate level of expertise to confirm that it is of an acceptable scientific standard.

# Reviewer report: Reviewer response for version 1

**DOI:** 10.5256/f1000research.193123.r461147

**Published:** 2026-03-13

**Authors:** Ayshwarya Subramanian, Adam Zuchowski

**Affiliations:** 1 Cornell University, Ithaca, new york, USA; 2 Biomedical Engineering, Cornell University College of Engineering (Ringgold ID: 70235), Ithaca, New York, USA

**Summary:** This manuscript presents SpotSweeper-py, a Python implementation of the SpotSweeper R package (PMID: **40481362**), a tool designed for spatially aware quality control of spatial transcriptomics data. By integrating spatial QC functionality within the Python scverse ecosystem widely used for single-cell omics analysis, the work fills a clear methodological gap for users working in Python-based workflows.

 The manuscript demonstrates the utility and scalability of SpotSweeper-py through applications to widely used spatial transcriptomics platforms, including Visium and VisiumHD. The software implementation is well documented, and the code is openly available. The package is user-friendly, with clear installation instructions and example usage that should facilitate adoption by the community.

 However, because the primary contribution of this manuscript is the reimplementation of an existing method from R into Python, Sections 2.1-2.3 would benefit from a more direct comparison with the original SpotSweeper R package. The current presentation largely reiterates the methodological contributions of the original paper rather than emphasizing what is new in the Python implementation. Benchmarking the two tools in terms of runtime, computational efficiency, and similarity of results would strengthen the manuscript. In addition, claims regarding the biological relevance and spatial context of detected local outliers would benefit from clearer definitions and supporting evidence.

**Major Concerns:**

 1. Claims regarding the biological and spatial context of local outliers The manuscript states that local outliers identified by SpotSweeper-py are “biologically meaningful” and “spatially contextualized” (page 5). However, these terms are not clearly defined within the manuscript, and no direct metrics or empirical evidence are presented to support these claims.

*Suggestion:* The authors could address this in 2 ways: explicitly cite and defer to the relevant analyses from the original SpotSweeper publication, clarifying that the evidence supporting these claims originates from prior work.

 OR provide a brief demonstration within the current manuscript, similar to Figure 2a-d in the original paper, showing that local thresholding preserves biologically meaningful spatial spots that would otherwise be removed by global QC thresholds.

 2. Side-by-side comparison and benchmarking against SpotSweeper R Because the goal of this work is to bring the functionality of SpotSweeper to the Python/scverse ecosystem, benchmarking against the existing R implementation would significantly strengthen the manuscript. Specifically, it would be helpful to know:

 - Whether the Python and R implementations produce consistent results on the same datasets.

 - How the tools compare in terms of runtime and computational resource usage.

 -Whether the Python implementation introduces improvements in scalability or integration with Python-based analysis workflows.

*Suggestion:* The original SpotSweeper paper reports a processing rate of approximately 1 second per 1,000 spots per metric. Including a comparable metric for SpotSweeper-py would provide a useful point of reference. Additionally, demonstrating that both implementations identify the same or highly similar sets of local outliers when applied to identical input datasets would strengthen confidence in the Python implementation.

****

**Minor Concerns:**

 (1) The manuscript uses a specific value of *k* when computing the median absolute deviation across neighboring spots. The rationale for this choice is not discussed. Providing guidance on how to select *k* for different spatial transcriptomics datasets or modalities would improve the practical usability of the method.

 (2) Section 4.1.4 introduces the constant 0.6745 in the calculation of the local z-score, but its origin is not explained. This constant corresponds to the inverse of the 75th percentile of the standard normal distribution and is used to scale the MAD to approximate the standard deviation. A brief explanation would help readers unfamiliar with robust statistics.

 (3) The violin plots shown in Figures 3, 7, and 9 contain dashed lines that are not described in the figure captions. The captions should clarify whether these lines represent the mean, median, or another summary statistic.

 (4) The comparison between global and local QC focuses on outliers uniquely identified by each approach. However, the manuscript does not discuss spots identified as outliers by both methods. Characterizing this shared set would provide useful context for understanding the degree of agreement between the approaches and could help clarify what additional information is provided by the local QC strategy.

 (5) Line 5 contains a likely typo: “inside” may have been intended to read “insight.”

Are the conclusions about the tool and its performance adequately supported by the findings presented in the article?

Partly

Is the rationale for developing the new software tool clearly explained?

Yes

Is the description of the software tool technically sound?

Yes

Are sufficient details of the code, methods and analysis (if applicable) provided to allow replication of the software development and its use by others?

Yes

Is sufficient information provided to allow interpretation of the expected output datasets and any results generated using the tool?

Yes

Reviewer Expertise:

single-cell technologies, computational biology

We confirm that we have read this submission and believe that we have an appropriate level of expertise to confirm that it is of an acceptable scientific standard, however we have significant reservations, as outlined above.

# Response

**Published:** 2026-09-09

**Authors:** Xingyi Chen

**Affiliations:** Applied Math & Statistics, Johns Hopkins University, Baltimore, Maryland, USA

Thank you for finding our proposed tool meaningful and addressing a clear methodological gap for users working in Python-based workflows. We have now carefully addressed your comments. Please find the point-by-point responses below.

 In our point-by-point response, we cite the reviewers’ unaltered comments in Italic typeface,  describe our responses and the resulting improvements to the revised manuscript in regular typeface.

**Major Concerns:**

*1. Claims regarding the biological and spatial context of local outliers The manuscript states that local outliers identified by SpotSweeper-py are “biologically meaningful” and “spatially contextualized” (page 5). However, these terms are not clearly defined within the manuscript, and no direct metrics or empirical evidence are presented to support these claims.*

*Suggestion: The authors could address this in 2 ways: explicitly cite and defer to the relevant analyses from the original SpotSweeper publication, clarifying that the evidence supporting these claims originates from prior work.*

*OR provide a brief demonstration within the current manuscript, similar to Figure 2a-d in the original paper, showing that local thresholding preserves biologically meaningful spatial spots that would otherwise be removed by global QC thresholds.*

 We thank the reviewer for this comment. We agree that the terms “biologically meaningful” and “spatially contextualized” were stronger than warranted by the analyses presented in this manuscript. We have therefore revised these statements throughout the manuscript to describe the observed results more directly in terms of spatially contiguous regions, neighborhood-relative deviations, and differences between local and global QC classifications. We also clarify in the Discussion that biological or technical interpretation of local outliers requires orthogonal validation and should not be inferred from QC metrics alone.

*2. Side-by-side comparison and benchmarking against SpotSweeper R Because the goal of this work is to bring the functionality of SpotSweeper to the Python/scverse ecosystem, benchmarking against the existing R implementation would significantly strengthen the manuscript. Specifically, it would be helpful to know:*

*- Whether the Python and R implementations produce consistent results on the same datasets.*

*- How the tools compare in terms of runtime and computational resource usage.*

*-Whether the Python implementation introduces improvements in scalability or integration with Python-based analysis workflows.*

*Suggestion: The original SpotSweeper paper reports a processing rate of approximately 1 second per 1,000 spots per metric. Including a comparable metric for SpotSweeper-py would provide a useful point of reference. Additionally, demonstrating that both implementations identify the same or highly similar sets of local outliers when applied to identical input datasets would strengthen confidence in the Python implementation.*

 We thank the reviewer for this suggestion. We have added a direct benchmark comparing SpotSweeper-py with the original SpotSweeper R implementation using the same Visium dataset and identical precomputed QC metrics, spatial coordinates, neighborhood size (k=36), outlier directions, cutoff, and single-worker execution. Across log total counts, number of detected genes, and percent mitochondrial counts, the local z-scores produced by the two implementations were highly concordant, with Pearson correlations of 1.0000, 1.0000, and 0.9999, respectively. These results demonstrate that SpotSweeper-py closely reproduces the neighborhood-relative QC signal generated by the original R implementation. 

 We also added a direct runtime comparison using repeated runs and report runtime normalized to seconds per 1,000 spots per metric. Under matched single-worker settings, SpotSweeper-py completed the local QC calculations at a comparable pace compared to the R implementation across all three metrics. These results are now presented in Figure 11 and Section 2.5. 

 In addition, we have clarified the computational advantages of the Python implementation in the manuscript. SpotSweeper-py operates directly on AnnData objects and integrates with the Python/scverse ecosystem, while our existing Visium HD analyses demonstrate practical scalability to 663,857 bins at 8-µm resolution and more than 10 million bins at 2-µm resolution. We have also added the benchmarking procedure to the Methods section. We believe these additions provide direct validation of consistency with the original SpotSweeper implementation while also demonstrating the computational performance and Python-native workflow integration of SpotSweeper-py.

**Minor Concerns:**

*(1) The manuscript uses a specific value of k when computing the median absolute deviation across neighboring spots. The rationale for this choice is not discussed. Providing guidance on how to select k for different spatial transcriptomics datasets or modalities would improve the practical usability of the method.*

 We thank the reviewer for this suggestion. We have expanded Sections 4.1.3 and 4.2.4 to provide both the rationale for the neighborhood sizes used in the manuscript and practical guidance for users. Following the original SpotSweeper framework, we now explain that neighborhood size can be defined according to spatial-neighborhood order for regular grids. A third-order neighborhood corresponds to k=36 for the Visium grid and k=48 for the Visium HD grid, which are the values used in our primary analyses. We clarify that k should be interpreted as a spatial-scale parameter rather than as an optimal constant. Smaller neighborhoods emphasize local deviations but can yield more variable local median and MAD estimates and more variable outlier calls. On the other hand, larger neighborhoods incorporate broader spatial context and may smooth highly localized deviations. We recommend starting with third-order neighborhood for regular Visium or Visium HD grids and adjusting k according to the platform geometry and spatial resolution. We also added a sensitivity analysis across neighborhood sizes and discussed the observed locality-stability tradeoff in Results and Discussion.

*(2) Section 4.1.4 introduces the constant 0.6745 in the calculation of the local z-score, but its origin is not explained. This constant corresponds to the inverse of the 75th percentile of the standard normal distribution and is used to scale the MAD to approximate the standard deviation. A brief explanation would help readers unfamiliar with robust statistics.*

 We thank the reviewer for this suggestion. We have added a brief explanation that 0.6745 corresponds approximately to the 75th percentile of the standard normal distribution and is used to scale the MAD to provide a robust analogue of the standard deviation under normality.

*(3) The violin plots shown in Figures 3, 7, and 9 contain dashed lines that are not described in the figure captions. The captions should clarify whether these lines represent the mean, median, or another summary statistic.*

 We thank the reviewer for noting this omission. We have updated the captions for Figures 3, 7, and 9 to clarify that the dashed horizontal lines indicate a local z-score of 0 and are included as reference lines.

*(4) The comparison between global and local QC focuses on outliers uniquely identified by each approach. However, the manuscript does not discuss spots identified as outliers by both methods. Characterizing this shared set would provide useful context for understanding the degree of agreement between the approaches and could help clarify what additional information is provided by the local QC strategy.*

 We thank the reviewer for this suggestion. We have added a quantitative characterization of the overlap between global and local QC classifications at the end of Section 2.1. In the Visium dataset, 26 spots were identified as outliers by both approaches, corresponding to 25.7% of locally flagged spots and 3.1% of globally flagged spots. We also report the numbers uniquely identified by each method and clarify that the shared set reflects agreement between the approaches, while locally unique outliers represent neighborhood-relative deviations not captured by global thresholds.

*(5) Line 5 contains a likely typo: “inside” may have been intended to read “insight.”*

 The typo has been fixed.

# Reviewer report: Reviewer response for version 1

**DOI:** 10.5256/f1000research.193123.r455887

**Published:** 2026-02-24

**Authors:** Patrick Murigu Kamau Njage

**Affiliations:** 1 Technical University of Denmark, Lyngby, Denmark

Page 3: revise the sentence “Local outliers are then flagged one or two sided based on user-defined cutoffs.”

 Page 3, Section 2.1 line 3: UMI- define this abbreviation at place of first mention in the manuscript.

 Page 4, line 2, last line: it is not clear how ground-truthing was performed to conclude that discarded regions were biologically meaningful. Biological meaning can only be inferred based on a validated comparison between the discarded regions and those from e.g. histological examination or other biological characterisation.

 Page 9: “This separation demonstrates that local QC captures spatially aware deviations that define technical artifacts at a high resolution, whereas global QC merely flags significant global shifts.”- this inference may be erroneous concerning what consists of technical artifacts or not. Basing this distinction on z-score distribution may lead to falsely flagging as an artifact of true but exceptional biologically meaningful data.

 Page 10 “ SpotSweeper-py reduced false positives…”- related to above, it is not clear if a golden standard was used to verify the results and conclude about false positives.

 Page 10: “Here, we demonstrated how to control bias in outlier detection…” it is not clear if bias metrics were used to clearly show that bias was controlled.

 Page 10: “The core function to detect local outliers, operates on a single anndata. AnnData object and expects the following

 components to be available in the AnnData object.” It seems like the repeat of anndata and split of the sentence was not intended.

 Section 4.1.3: This section quotes k=36 as a default. The study, however, uses other values such as k=48 in section 2.3. This raises the question if the results of the study are not influenced and only specific for the by values of k used. How about other values of k such as 20, 40 etc?  

 Below is a structured **reviewer report** for the manuscript:

**Chen X, Totty M, Hicks S. *Spotsweeper-py: spatially-aware quality control metrics for spatial omics data in the Python ecosystem***

**Reviewer Report**

**General Assessment**

 This manuscript presents *SpotSweeper-py*, a Python implementation of the previously published SpotSweeper framework for spatially-aware quality control (QC) in spatial transcriptomics data . The work addresses a meaningful gap by translating robust local QC methods into the Python/scverse ecosystem, where spatial transcriptomics workflows are increasingly standardized. The manuscript is clearly structured, and the use cases on Visium and VisiumHD datasets effectively demonstrate computational feasibility and practical integration with AnnData workflows.

 The implementation appears well engineered, reproducible, and documented. The methodological sections are technically sound and transparent. However, several interpretive statements require clarification or more cautious framing, particularly regarding claims about “false positives,” “technical artifacts,” and “bias control.” Some wording and definitional clarifications are also needed.

 Overall, the work is valuable and likely to be impactful after revision.

**Major Comments**

**1. Interpretation of Local QC as Defining Technical Artifacts (Page 9)**

 The statement:

 “This separation demonstrates that local QC captures spatially aware deviations that define technical artifacts at a high resolution…” (Page 9)

 is interpretatively strong and potentially overstated.

 While the clear separation of local z-score distributions (Figure 9B) supports that the method detects spatially localized deviations, it does not establish that these deviations correspond to *technical artifacts*. Exceptional or rare biological phenomena (e.g., necrotic niches, metabolic hotspots, tumor microenvironment gradients) may also appear as neighborhood-level deviations.

**Recommendation:**

 Rephrase to reflect statistical detection of spatial deviations rather than definitive identification of technical artifacts. In addition, discuss more explicitly that confirmation requires orthogonal biological validation.

**2. False Positive Claims Without a Gold Standard (Page 10)**

 The manuscript states:

 “SpotSweeper-py reduced false positives…” (Page 10)

 However, no gold-standard annotations (e.g., histological validation, pathologist labeling, imaging-based assessment, or spike-in controls) are presented to define what constitutes a false positive.

 Reduced flagging relative to global thresholds does not necessarily imply fewer false positives — it may instead reflect reduced sensitivity. The manuscript currently conflates reduced flagging rate with error reduction.

**Recommendation:**

 Either:

- Provide evidence of external validation (histology alignment, manual annotation comparison, biological enrichment tests), or
- Rephrase to a more cautious interpretation:

**3. Ground-Truthing and Biological Meaning (Page 4)**

 The manuscript suggests that globally discarded regions are “likely biologically meaningful” (Page 4) , but it is unclear how this conclusion was established.

 Biological meaningfulness cannot be inferred without validated comparison, such as:

- Histological overlays
- Marker gene expression consistency
- Pathologist annotations
- Morphological segmentation
- Comparative QC with orthogonal spatial technologies

**Recommendation:**

 Clarify what constitutes “biologically meaningful.” If no external validation is available, soften language accordingly:

 E.g. “...large contiguous regions that may correspond to intact tissue architecture.”

**4. Claim of Bias Control (Page 10)**

 The manuscript states:

 “Here, we demonstrated how to control bias in outlier detection…”

 However, no explicit bias metrics are defined, quantified, or benchmarked. While the use of medians, MAD, and log transformations reduces influence of extreme values, this represents robustness, not explicitly measured bias control.

**Recommendation:**

 Rephrase to:

 “Here, we demonstrate how robust and neighborhood-aware statistics can mitigate the influence of extreme values in outlier detection.”

 Alternatively, quantify bias if formally evaluated.

**5. Sensitivity to k (Neighborhood Size)**

 Section 4.1.3 states that the default is k = 36 , while Sections 2.2–2.3 use k = 48 for VisiumHD datasets. No sensitivity analysis is presented to evaluate robustness of findings across k ∈ {20, 30, 40, 48, etc.}.

 Since neighborhood size directly controls bias–variance tradeoff:

- Smaller k increases variance and detection sensitivity
- Larger k increases smoothing and may mask local anomalies

**Recommendation:**

 Include:

- Sensitivity analysis for several k values
- Plot showing proportion flagged vs k
- Discussion of stability of spatial patterns across k

Even a brief supplementary analysis would strengthen reproducibility and generalizability.

**Minor Comments**

**1. Sentence Clarification (Page 3)**

 Original:

 “Local outliers are then flagged one or two sided based on user-defined cutoffs.”

 Suggested revision:

 “Local outliers are then flagged as one-sided or two-sided based on user-defined z-score cutoffs.”

**2. Define UMI (Page 3, Section 2.1)**

 “UMI” should be defined at first use as:

 Unique Molecular Identifier (UMI)

**3. Typographical / Sentence Fragment (Page 10)**

 Original:

 “The core function to detect local outliers, operates on a single anndata. AnnData object…”

 Correct to:

 “The core function to detect local outliers operates on a single AnnData object…”

**4. Clarify “mis-flagged” Language (Page 6; Figure 4)**

 The manuscript states that spots are “mis-flagged” by global QC .

 Unless validated, this should be softened to:

 “flagged by global QC but not by local QC”

**Minor comments**

1. **Explicit Limitations Section**
  The discussion should more clearly acknowledge:
  - No gold standard labeling
  - Assumption of uniform spatial density
  - Possible oversensitivity in edge regions
  - Sensitivity to anisotropic tissue structure
2. **Compare with Alternative Methods**
  Given references to other QC tools (e.g., SpatialQC, Sprod), a short comparison table would strengthen positioning if possible.
3. **Computational Complexity Characterization**
  Runtime scaling is described narratively; including O(n log n) or similar computational complexity analysis would improve technical clarity.

Are the conclusions about the tool and its performance adequately supported by the findings presented in the article?

Partly

Is the rationale for developing the new software tool clearly explained?

Yes

Is the description of the software tool technically sound?

Yes

Are sufficient details of the code, methods and analysis (if applicable) provided to allow replication of the software development and its use by others?

Yes

Is sufficient information provided to allow interpretation of the expected output datasets and any results generated using the tool?

Yes

Reviewer Expertise:

Genomic epidemiology. Use of data science and statistics for infectious disease modelling.

I confirm that I have read this submission and believe that I have an appropriate level of expertise to confirm that it is of an acceptable scientific standard, however I have significant reservations, as outlined above.

# Response

**Published:** 2026-09-09

**Authors:** Xingyi Chen

**Affiliations:** Applied Math & Statistics, Johns Hopkins University, Baltimore, Maryland, USA

Thank you for finding our proposed tool meaningful and addressing a clear methodological gap for users working in Python-based workflows. We have now carefully addressed your comments. Please find the point-by-point responses below. 

 In our point-by-point response, we cite the reviewers’ unaltered comments in Italic typeface,  describe our responses and the resulting improvements to the revised manuscript in regular typeface.

**Major Concerns:**

*1. Interpretation of Local QC as Defining Technical Artifacts (Page 9)*

*The statement: “This separation demonstrates that local QC captures spatially aware deviations that define technical artifacts at a high resolution…” (Page 9) is interpretatively strong and potentially overstated. While the clear separation of local z-score distributions (Figure 9B) supports that the method detects spatially localized deviations, it does not establish that these deviations correspond to technical artifacts. Exceptional or rare biological phenomena (e.g., necrotic niches, metabolic hotspots, tumor microenvironment gradients) may also appear as neighborhood-level deviations.*

*Recommendation: Rephrase to reflect statistical detection of spatial deviations rather than definitive identification of technical artifacts. In addition, discuss more explicitly that confirmation requires orthogonal biological validation.*

 We thank the reviewer for this important distinction. We agree that separation of local z-score distributions demonstrates detection of neighborhood-relative spatial deviations but does not establish that these deviations are necessarily technical artifacts. We have therefore revised the statement in Section 2.3 to describe these observations as neighborhood-relative deviations and explicitly note that they may also reflect rare or spatially restricted biological phenomena. We have additionally expanded the Discussion to emphasize that SpotSweeper-py outliers are statistical QC indicators and that orthogonal evidence, such as histology or biological annotations, should be considered before interpreting flagged regions as technical artifacts or removing them from downstream analyses.

*2. False Positive Claims Without a Gold Standard (Page 10)*

*The manuscript states: “SpotSweeper-py reduced false positives…” (Page 10) However, no gold-standard annotations (e.g., histological validation, pathologist labeling, imaging-based assessment, or spike-in controls) are presented to define what constitutes a false positive. Reduced flagging relative to global thresholds does not necessarily imply fewer false positives — it may instead reflect reduced sensitivity. The manuscript currently conflates reduced flagging rate with error reduction.*

*Recommendation: Either: Provide evidence of external validation (histology alignment, manual annotation comparison, biological enrichment tests), or Rephrase to a more cautious interpretation.*

 We thank the reviewer for this clarification. We agree that, in the absence of an external gold standard, differences in outlier flagging rates cannot be interpreted directly as differences in false-positive rates. We have therefore removed language stating that SpotSweeper-py “reduces false positives” and revised the manuscript to describe the observed differences in terms of outlier frequency, spatial localization, and retention of spatially homogeneous regions. We also now explicitly note that the lack of histological or other orthogonal ground-truth annotations prevents direct estimation of false-positive and false-negative rates.

*3. Ground-Truthing and Biological Meaning (Page 4)*

*The manuscript suggests that globally discarded regions are “likely biologically meaningful” (Page 4) , but it is unclear how this conclusion was established. Biological meaningfulness cannot be inferred without validated comparison, such as: histological overlays; marker gene expression consistency; pathologist annotations; morphological segmentation; comparative QC with orthogonal spatial technologies*

*Recommendation: Clarify what constitutes “biologically meaningful.” If no external validation is available, soften language accordingly: E.g. “...large contiguous regions that may correspond to intact tissue architecture.”*

 We thank the reviewer for this point. We agree that biological meaningfulness cannot be established from QC metrics alone without orthogonal validation. We have therefore softened this language throughout the manuscript. In particular, we now describe these regions as large, spatially contiguous or homogeneous regions that may correspond to intact tissue architecture, rather than asserting that they are biologically meaningful. We also clarify that external validation, such as histology or biological annotations, would be required to establish their biological interpretation.

*4. Claim of Bias Control (Page 10)*

*The manuscript states: “Here, we demonstrated how to control bias in outlier detection…” However, no explicit bias metrics are defined, quantified, or benchmarked. While the use of medians, MAD, and log transformations reduces influence of extreme values, this represents robustness, not explicitly measured bias control.*

*Recommendation: Rephrase to: “Here, we demonstrate how robust and neighborhood-aware statistics can mitigate the influence of extreme values in outlier detection.” Alternatively, quantify bias if formally evaluated.*

 We thank the reviewer for this distinction. We agree that our analyses evaluate robustness rather than formally quantified bias. We have therefore revised the text to state that robust and neighborhood-aware statistics can mitigate the influence of extreme values in outlier detection, rather than claiming explicit bias control.

*5. Sensitivity to k (Neighborhood Size)*

*Section 4.1.3 states that the default is k = 36 , while Sections 2.2–2.3 use k = 48 for VisiumHD datasets. No sensitivity analysis is presented to evaluate robustness of findings across k ∈ {20, 30, 40, 48, etc.}. Since neighborhood size directly controls bias–variance tradeoff: Smaller k increases variance and detection sensitivity; Larger k increases smoothing and may mask local anomalies*

*Recommendation: Include: Sensitivity analysis for several k values; Plot showing proportion flagged vs k; Discussion of stability of spatial patterns across k. Even a brief supplementary analysis would strengthen reproducibility and generalizability.*

 Thank you for this suggestion. We agree that the original manuscript did not sufficiently explain the different neighborhood sizes used for Visium and Visium HD or evaluate sensitivity to this parameter. We have now clarified that k=36 and k=48 correspond to the same conceptual neighborhood scale - a third-order spatial neighborhood - on the hexagonal Visium and square Visium HD grids following the original SpotSweeper framework. We also added a new sensitivity analysis in Section 2.4 and Figure 10. 

 For the Visium dataset, we evaluated first- through fifth-order neighborhoods, corresponding to k=6,18,36,60,90. We examined (i) the proportion of spots flagged across neighborhood sizes, (ii) Jaccard similarity of outlier calls with the manuscript setting of k=36, and (iii) the spatial distribution of log-total-count outliers for nearby neighborhood sizes k=18,36,60. Very small neighborhoods produced substantially more outlier calls, whereas the overall proportion flagged was more stable at larger neighborhood sizes. The exact identities of flagged spots remained dependent on k; however, the major spatial locations of detected anomalies were qualitatively similar across nearby neighborhood sizes. We then repeated the sensitivity analysis for the Visium HD 8-µm dataset using neighborhood sizes k=8,24,48,80,120 and observed a similar overall dependence on neighborhood scale. 

 We now discuss this locality-stability tradeoff in the Discussion and retain the third-order neighborhoods k=36 for Visium and k=48 for Visium HD as practical defaults.

**Minor Concerns:**

*1. Explicit Limitations Section*

*The discussion should more clearly acknowledge: No gold standard labeling; Assumption of uniform spatial density; Possible oversensitivity in edge regions; Sensitivity to anisotropic tissue structure*

 We thank the reviewer for this suggestion. We have expanded the limitations discussion to explicitly acknowledge the absence of gold-standard artifact annotations, the assumption of relatively uniform spatial density, potential edge effects arising from asymmetric neighborhoods near tissue boundaries, and sensitivity to anisotropic or irregular tissue structure. We also clarify that these factors may influence local z-scores and outlier calls and should be considered when interpreting SpotSweeper-py results.

*2. Compare with Alternative Methods*

*Given references to other QC tools (e.g., SpatialQC, Sprod), a short comparison table would strengthen positioning if possible.*

 We thank the reviewer for this suggestion. We agree that clearer positioning relative to existing spatial transcriptomics QC tools is useful. Rather than adding a quantitative comparison table, we expanded the Introduction to briefly distinguish SpotSweeper-py from related approaches including SpotClean, Sprod, and SpatialQC. Specifically, we now clarify that these methods address tasks such as contamination correction, denoising, or broader automated QC, while SpotSweeper-py focuses on local outlier detection using standard sequencing-derived QC metrics and provides this functionality within the Python/scverse ecosystem.

*3. Computational Complexity Characterization*

*Runtime scaling is described narratively; including O(n log n) or similar computational complexity analysis would improve technical clarity.*

 We thank the reviewer for this suggestion. We have added a computational complexity characterization to the Methods section at the end of 4.1.3. Because SpotSweeper-py relies on scikit-learn nearest-neighbor search with automatic algorithm selection, complexity depends on the selected backend. Tree-based neighborhood construction is typically approximately O(n log n) in low-dimensional spatial coordinates.

*Minor Line-edits:*

- *Sentence Clarification (Page 3) Original: “Local outliers are then flagged one or two sided based on user-defined cutoffs.” Suggested revision: “Local outliers are then flagged as one-sided or two-sided based on user-defined z-score cutoffs.”*
- *Define UMI (Page 3, Section 2.1) “UMI” should be defined at first use as: Unique Molecular Identifier (UMI) *
- *Typographical / Sentence Fragment (Page 10) Original: “The core function to detect local outliers, operates on a single anndata. AnnData object…” Correct to: “The core function to detect local outliers operates on a single AnnData object…” *
- *Clarify “mis-flagged” Language (Page 6; Figure 4) The manuscript states that spots are “mis-flagged” by global QC . Unless validated, this should be softened to: “flagged by global QC but not by local QC”*

We thank the reviewer for the line-edits. We have modified corresponding sentences throughout the manuscript.
